# Price and Variability in Hospital Charges for Generic Indomethacin Suppositories

**DOI:** 10.1001/jamanetworkopen.2024.35528

**Published:** 2024-09-26

**Authors:** Noah M. Feder, Inmaculada Hernandez, William B. Feldman, Mathavan Murali, Aaron S. Kesselheim, B. Joseph Elmunzer, Walid F. Gellad

**Affiliations:** 1 Center for Pharmaceutical Policy and Prescribing, University of Pittsburgh, Pittsburgh, Pennsylvania; 2 UCSD Skaggs School of Pharmacy and Pharmaceutical Sciences, La Jolla, California; 3 Program On Regulation, Therapeutics, And Law, Division of Pharmacoepidemiology and Pharmacoeconomics, Department of Medicine, Brigham and Women’s Hospital and Harvard Medical School, Boston, Massachusetts; 4 Division of Pulmonary and Critical Care Medicine, Department of Medicine, Brigham and Women’s Hospital and Harvard Medical School, Boston, Massachusetts; 5 Division of Gastroenterology and Hepatology, Medical University of South Carolina, Charleston

## Abstract

This cross-sectional study examines how list prices for indomethacin have changed since 2019 and characterizes variation in hospital charges for the drug, including gross charges and discounted cash prices.

## Introduction

Indomethacin, a nonsteroidal anti-inflammatory drug approved by the Food and Drug Administration (FDA) in the 1950s, was introduced in a suppository formulation in 1984. Made by one manufacturer until recently, indomethacin suppository prices have dramatically increased since a 2012 randomized clinical trial found that prophylactic administration of a 100-mg dose decreased pancreatitis risk by 50% after endoscopic retrograde cholangiopancreatography (ERCP).^1,2,3^

Medicare beneficiaries undergoing ERCP may be billed the full cost of periprocedural indomethacin, exposing them to the drug’s rising price. This is because the drug is excluded from coverage under Medicare Part B, as it is considered a self-administered product, and is excluded from coverage under Medicare Part D because it is administered in a hospital setting.^4^

Our previous analysis documented the rising price for indomethacin suppositories,^2^ but Medicare beneficiaries face even higher prices because hospital charges for pharmaceuticals can far exceed list prices. We sought to understand how list prices for indomethacin have changed since our prior report^2^ and to describe variation in hospital charges for the drug, including gross charges (chargemaster prices) and discounted cash prices (prices for self-pay patients).

## Methods

The University of Pittsburgh institutional review board determined that this cross-sectional study was exempt from review and did not require informed consent because it was not human participants research. We followed the STROBE reporting guideline.

We obtained the wholesale acquisition cost (list price) for indomethacin suppositories from Navlin for November 2008 to March 2024. We then obtained a list of US hospitals from the 2021 Hospital Provider Cost Report.^5^ For each hospital, we obtained publicly available standard charge files (from July 2023 to January 2024). We then searched in descending bed-size order until we found 50 charge files out of 95 total hospitals (eAppendix in Supplement 1) that included both the gross charge and a discounted cash price for indomethacin suppositories. We excluded hospitals that had more than one entry for rectal indomethacin (n = 2) or did not contain both a cash and gross price (n = 6). For each hospital, we reported the gross charge and the discounted cash price for a 100-mg dose (2 50-mg suppositories). Statistical analysis was performed in March 2024 using Stata SE 18 (StataCorp).

## Results

**Figure 1.**
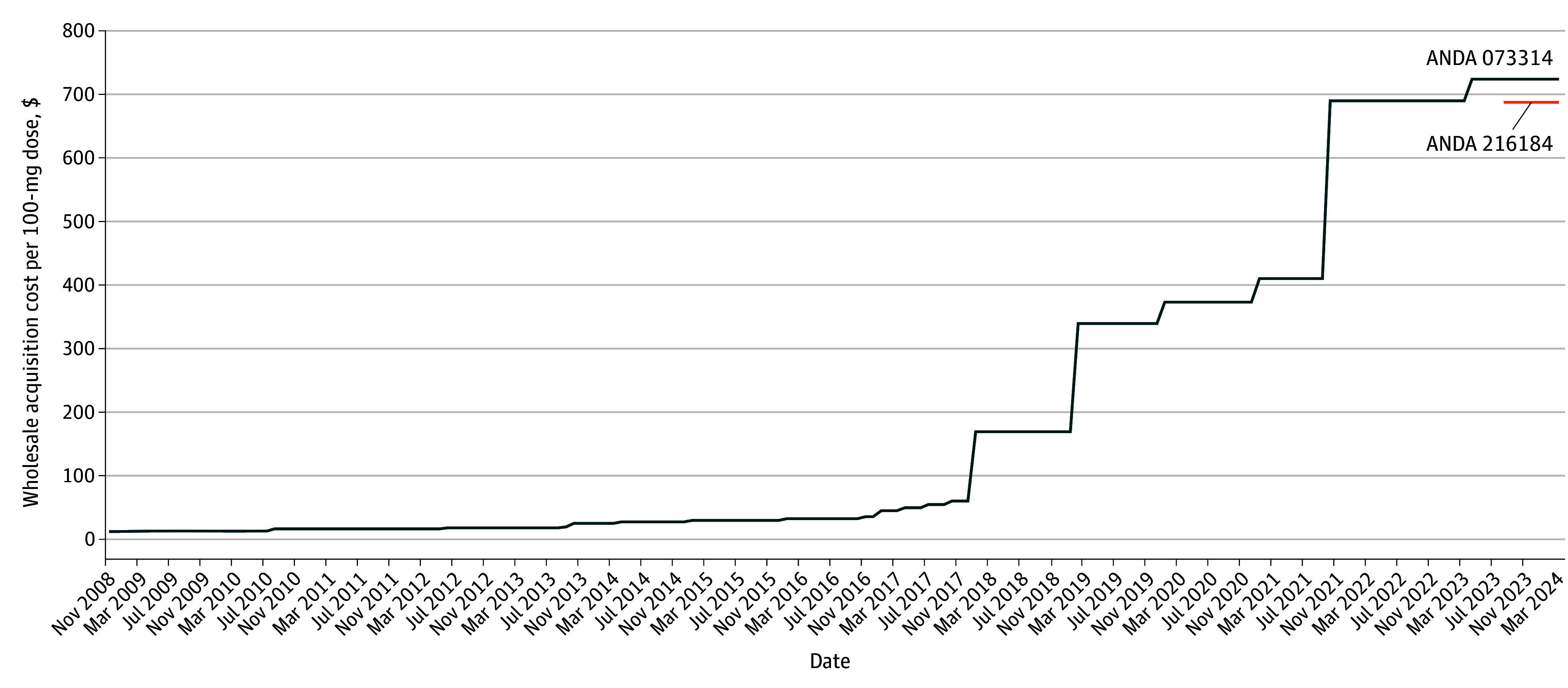
Wholesale Acquisition Cost for a 100-mg Dose of Rectal Indomethacin From November 2008 Through March 2024 In August 2023, a second generic manufacturer entered the market with an indomethacin suppository (orange line). ANDA indicates Abbreviated New Drug Application number for the generic product.

The list price for a 100-mg dose of rectal indomethacin increased from $13.20 in 2008 to $61.09 in 2017, and then steeply increased further to $723.81 by 2023. One additional manufacturer of indomethacin entered the market in August 2023, offering a list price of $687.62 (Figure 1).

**Figure 2.**
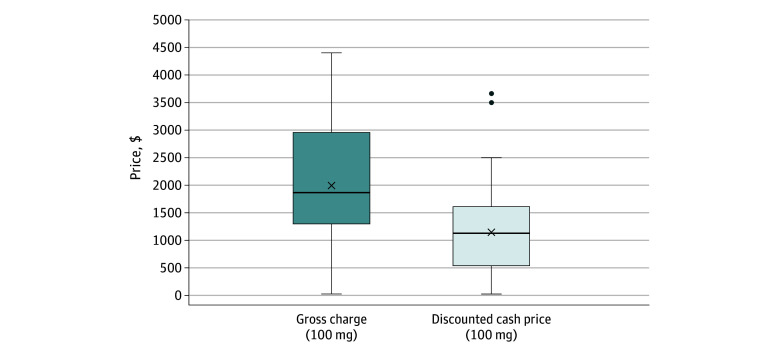
Variation in Hospital Gross Charge and Discounted Cash Price for a 100-mg Dose of Rectal Indomethacin Across 50 of the Largest US Hospitals by Bed Size Box plots represent 25th to 75th percentile with horizontal line indicating median and x indicating mean. Data points outside of the box plots are outliers. Data collected from July 2023 to January 2024.

Of the 50 hospitals in our final cohort, the median (IQR) gross charge for a 100-mg dose was $1867.34 ($1297.90-$2949.20), and the median (IQR) discounted cash price was $1126.88 ($538.20-$1594.00) (Figure 2).

## Discussion

Seventy years after indomethacin’s approval and 40 years after its introduction in suppository form, the list price for a 100-mg dose is more than $720, representing a greater than 50-fold increase compared with 15 years ago. Despite not being protected by FDA-listed patents or other known exclusivities, rectal indomethacin had a single manufacturer until very recently, which likely explains the substantial price hikes.

A limitation of our study is that our findings may not generalize to indomethacin administered at smaller hospitals or clinics or to patients covered by private insurance. Nonetheless, high hospital charges for the drug represent a clear financial burden for Medicare patients undergoing ERCP, whether they pay discounted cash prices or gross charges, with wide geographic variability. Some hospitals have resorted to compounding diclofenac, another anti-inflammatory drug,^3^ or asking patients to fill indomethacin prescriptions at outpatient pharmacies (paid via Part D) and bring the drug with them in a cooler to the ERCP.

Government regulators should investigate why there was only one manufacturer of this generic product until very recently. Legislators should consider ways to improve affordability, including expanding the list of exceptions for self-administered drugs covered under Medicare Part B and considering public manufacturing.^6^

## Notes

Supplement 1. **eAppendix.** Supplemental Materials

Supplement 2.
Data Sharing Statement
