# Supplementary material for: Price and Variability in Hospital Charges for Generic Indomethacin Suppositories
Source: JAMA Netw Open. 2024 Sep 26;7(9):e2435528. doi: 10.1001/jamanetworkopen.2024.35528 (PMC11428000; doi:10.1001/jamanetworkopen.2024.35528)
Supplement: Supplement 1. — eAppendix. Supplemental Materials [file jamanetwopen-e2435528-s001.pdf]

Supplemental Online Content

Feder NM, Hernandez I, Feldman WB, et al. Price and variability in hospital charges for generic indomethacin suppositories. *JAMA Netw Open.* 2024;7(9):e2435528. doi:10.1001/jamanetworkopen.2024.35528

eAppendix. Supplementary Materials

This supplemental material has been provided by the authors to give readers additional information about their work.

© 2024 Feder NM et al. *JAMA Network Open.*

eAppendix. Supplementary Materials

Inclusion/Exclusion of hospitals: To identify the 50 hospitals in our final cohort, we reviewed charge data from a total of 95 hospitals. Of the 95 hospitals, 93 (98%) had a standard charge file on their website, and 58 (61%) had entries for indomethacin suppositories. We then excluded eight (8%) hospitals that had more than one entry for indomethacin suppositories or did not contain a cash price. The final list of included and excluded hospitals and their associated bed-sizes is in **eTable 1** and **eTable 2**, respectively, below. Median bed sizes were similar between included and excluded hospitals. Excluded hospitals were more likely to be in Florida, NY, and CA (**Figure 1** below).

© 2024 Feder NM et al. *JAMA Network Open*.

eTable 1: List of included hospitals

Included		
**Hospital Name**	**State Code**	**Number of Beds**
METHODIST HOSPITAL	TX	1,742
NYU LANGONE HOSPITALS	NY	1,571
CLEVELAND CLINIC HOSPITAL	OH	1,326
YALE-NEW HAVEN HOSPITAL	CT	1,306
METHODIST H/C MEMPHIS HOSPT.	TN	1,295
BARNES-JEWISH HOSPITAL	MO	1,273
INDIANA UNIVERSITY HEALTH	IN	1,265
LEHIGH VALLEY	PA	1,203
CHRISTIANA CARE HEALTH SYSTEM	DE	1,180
LONG ISLAND JEWISH MEDICAL CENTER	NY	1,166
MAYO CLINIC HOSPITAL ROCHESTER	MN	1,143
UPMC - PRESBYTERIAN SHADYSIDE	PA	1,141
CAROLINAS MEDICAL CENTER	NC	1,134
UNIVERSITY OF ALABAMA HOSPITAL	AL	1,129
MEMORIAL HERMANN TEXAS MEDICAL CNTR	TX	1,076
WILLIAM BEAUMONT HOSPITAL- ROYAL OAK	MI	1,026
OCHSNER CLINIC FOUNDATION	LA	1,024
THE JOHNS HOPKINS HOSPITAL	MD	999
UF HEALTH SHANDS	FL	965
UNIV OF MI HOSPITALS & HLTH CTRS	MI	951
UNIVERSITY OF KANSAS HOSPITAL	KS	940
HUNTSVILLE HOSPITAL	AL	904
TAMPA GENERAL HOSPITAL	FL	898
TEXAS CHILDRENS HOSPITAL	TX	861
BAPTIST MEM HOSPITAL MEMPHIS	TN	848
UNIVERSITY OF MARYLAND MED SYS	MD	846
MERCY HOSPITAL - ST. LOUIS	MO	843
SHARP MEMORIAL HOSPITAL	CA	832
THE MOSES H. CONE MEMORIAL HOSPITAL	NC	822
MEDICAL CITY DALLAS	TX	819
NORTH CAROLINA BAPTIST HOSPITAL	NC	815
OU MEDICAL CENTER	OK	812
BRIGHAM AND WOMENS HOSPITAL	MA	812
THE UNIVERSITY OF TEXAS MEDICAL BR.	TX	810
DALLAS CO. HOSP. DIST.	TX	801
INOVA FAIRFAX HOSPITAL	VA	794
COXHEALTH	MO	791
PARKVIEW HOSPITAL	IN	791
WILLIS-KNIGHTON HEALTH SYSTEMS	LA	790
UOFL HEALTH-LOUISVILLE	KY	789
NORTH SHORE UNIVERSITY HOSPITAL	NY	782
OUR LADY OF THE LAKE RMC	LA	777
MEMORIAL REGIONAL HOSPITAL	FL	772
SOUTH TEXAS HEALTH SYSTEM	TX	770
RIVERSIDE METHODIST HOSPITAL	OH	750
ALBANY MEDICAL CENTER HOSPITAL	NY	743
MISSION HOSPITAL INC	NC	733
UNIVERSITY OF IOWA HOSP & CLINICS	IA	732
GHS GREENVILLE MEMORIAL HOSPITAL	SC	728
MERCY HOSPITAL SOUTH	MO	720
	Q1	791
	Median	847
	Q3	1133

eTable 2: List of excluded hospitals

Excluded		
**Hospital Name**	**State Code**	**Number of Beds**
ADVENTHEALTH ORLANDO	FL	2,791
NEW YORK PRESBYTERIAN HOSPITAL	NY	2,334
ORLANDO HEALTH	FL	1,600
JACKSON MEMORIAL	FL	1,504
NORTON HOSPITALS INC	KY	1,465
BAPTIST HEALTH SYSTEM	TX	1,451
MONTEFIORE MEDICAL CENTER	NY	1,426
ST. JOSEPHS HOSPITAL	FL	1,382
SPECTRUM HEALTH HOSPITALS	MI	1,148
MOUNT SINAI HOSPITAL	NY	1,080
VANDERBILT UNIVERSITY MEDICAL CENTER	TN	1,056
DUKE UNIVERSITY HOSPITAL	NC	1,048
THE OHIO STATE UNIVERSITY HOSPITAL	OH	1,016
METROPOLITAN STATE HOSPITAL	CA	1,004
THE METHODIST HOSPITAL	TX	1,003
HOSPITAL OF THE UNIV OF PENNA	PA	1,003
MASSACHUSETTS GENERAL HOSPITAL	MA	995
UNIVERSITY HOSPITAL	KY	962
KALEIDA HEALTH	NY	954
AURORA HEALTH CARE METRO INC.	WI	915
FORSYTH MEMORIAL HOSPITAL INC	NC	912
NORTHWESTERN MEMORIAL HOSPITAL	IL	901
SAINT FRANCIS HOSPITAL	OK	883
BAPTIST MEDICAL CENTER	FL	882
CEDARS-SINAI MEDICAL CENTER	CA	882
MIAMI VALLEY HOSPITAL	OH	878
THOMAS JEFFERSON UNIV. HOSPITAL	PA	868
PITT COUNTY MEMORIAL HOSPITAL	NC	847
BAYLOR UNIVERSITY MEDICAL CTR	TX	844
BAPTIST HOSPITAL	FL	838
CHARLESTON AREA MEDICAL CENTER INC.	WV	813
SANTA CLARA VALLEY MEDICAL CENTER	CA	812
UNIVERSITY OF NORTH CAROLINA HOSP.	NC	811
UCSF MEDICAL CENTER	CA	809
ASCENSION ST. VINCENT HOSPITAL	IN	799
BAPTIST HEALTH MEDICAL CENTER - LR	AR	785
LAKELAND REGIONAL MED CTR	FL	774
LEE MEMORIAL HOSPITAL	FL	768
TEMPLE UNIVERSITY HOSPITAL	PA	761
COMMUNITY REGIONAL MEDICAL CENTER	CA	761
STRONG MEMORIAL HOSPITAL	NY	757
NORTH SHORE MEDICAL CENTER AND FMC	FL	748
STONY BROOK UNIVERSITY HOSPITAL	NY	725
MEDICAL UNIVERSITY OF SOUTH CAROLINA	SC	722
NORTHSIDE HOSPITAL	GA	721
	Q1	809
	Median	883
	Q3	1048

© 2024 Feder NM et al. *JAMA Network Open*.

Figure 1: Count of hospitals included (orange) and excluded (blue) from analysis, by state

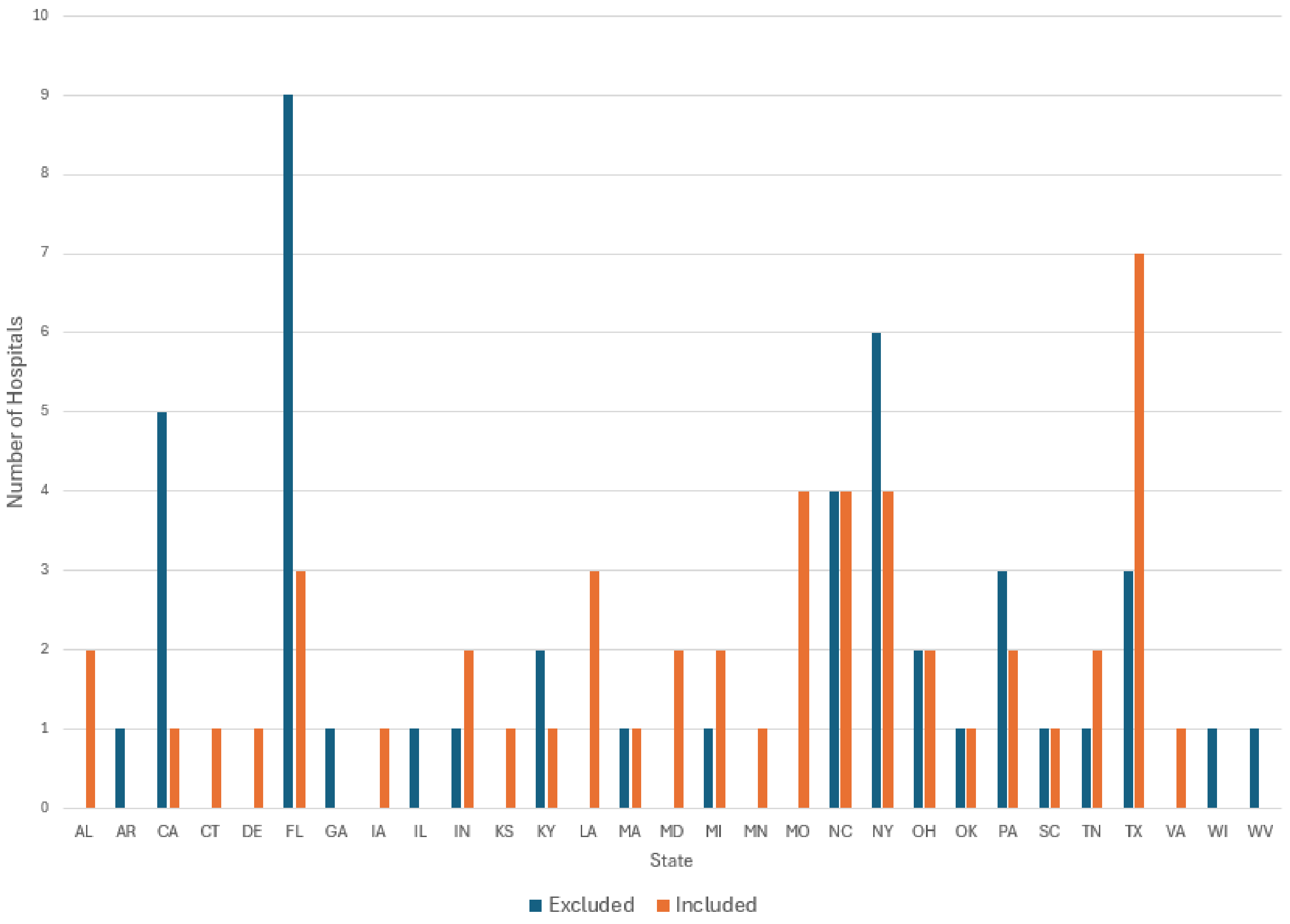

© 2024 Feder NM et al. *JAMA Network Open*.
