# Supplementary material for: Price and Variability in Hospital Charges for Generic Indomethacin Suppositories
Source: JAMA Netw Open. 2024 Sep 26;7(9):e2435528. doi: 10.1001/jamanetworkopen.2024.35528 (PMC11428000; doi:10.1001/jamanetworkopen.2024.35528)
Supplement: Supplement 2. — Data Sharing Statement [file jamanetwopen-e2435528-s002.pdf]

Data Sharing Statement

Feder. Price and Variability in Hospital Charges for Generic Indomethacin Suppositories. *JAMA Netw Open*. Published September 26, 2024. doi:10.1001/jamanetworkopen.2024.35528

Data
Data available: No

Additional Information
Explanation for why data not available: Hospital charge data are publicly available. A dataset can be made available for other researcher use if requested. The drug pricing data are proprietary and cannot be shared
